# Clinico-Epidemiological Profile of Breakthrough COVID-19 Infection among Vaccinated Beneficiaries from a COVID-19 Vaccination Centre in Bihar, India

**DOI:** 10.4314/ejhs.v32i1.3

**Published:** 2022-01

**Authors:** Chandra Mani Singh, Prashant Kumar Singh, Bijaya Nanda Naik, Sanjay Pandey, Santosh Kumar Nirala, Prabhat Kumar Singh

**Affiliations:** 1 Department of Community and Family Medicine, All India Institute of Medical Sciences (AIIMS), Patna

**Keywords:** SARS CoV-2, Breakthrough COVID infections, Vaccination, COVID-19, Covaxin, India

## Abstract

**Background:**

When the whole world is fighting in an unprecedented pace against COVID-19 pandemic, the breakthrough COVID infections poise to dampen the rapid control of the same. We carried out this project with two objectives; first, to estimate the proportion of breakthrough COVID-19 infection among completely vaccinated individuals and second, to study the clinico-epidemiological profile of breakthrough COVID-19 infections among them.

**Methods:**

This cross-sectional analytical study was conducted among 2703 fully vaccinated individuals from AIIMS, Patna COVID Vaccination Centre (CVC), Bihar, India. The participants were selected randomly using a systematic sampling technique from the list of beneficiaries maintained at the CVC. Telephonic interviews were made to collect the information by trained data collectors.

**Results:**

A total of 274 fully vaccinated beneficiaries [10.1% (95% CI: 9.1%, 11.4%)] were diagnosed with breakthrough COVID-19 infection. The infections were more among males (10.4%) and the individuals aged ≤29 years (12.5%). The beneficiary categories, the healthcare-worker and the frontline-worker, were identified as predictors of the breakthrough COVID infections. Only one in three participants had adopted adequate COVID appropriate behaviour following the full vaccination. The majority of the breakthrough infections occurred during the second wave of COVID-19. The majority of the individuals with breakthrough infections were asymptomatic and no death was reported among them.

**Conclusion:**

One in every ten fully vaccinated individuals can get the breakthrough COVID infections. The healthcare-worker and the frontline-worker had independent risk of getting the breakthrough infections. Very few with breakthrough infections were serious and no death was reported among them.

## Introduction

The Coronavirus disease 2019 (COVID-19), the global pandemic caused by Severe Acute Respiratory Syndrome Corona virus -2 (SARS CoV-2), has devastating effects on India with 2,22,96,414 patients and 2,42,362 deaths being reported as on 9^th^ May 2021 (1,2). A herd immunity level of 60%–70% is crucial to control the spread of infections during this pandemic, and that vaccine remains the most crucial and effective weapon for the same (3–6). All the countries around the world started the COVID-19 vaccination campaign during late 2020 and early 2021. The Government of India launched world biggest COVID vaccination campaign on 16^th^ January 2021, with two vaccines [Covaxin (BBV152), Indigenous, Bharat Biotech Ltd.; Covishield (ChAdOx1 nCoV — 19), Serum Institute of India with technology transfer from Oxford University and AstraZeneca] in phased manner to prevent spread of SARS CoV-2 infection and control the pandemic. The operational guidelines for COVID-19 were issued by Government of India on 28^th^ December 2020 (7). The two vaccines used in India have also demonstrated very good efficacy against COVID infection (8,9). The Covaxin, administered with two doses four weeks apart, is reported to have overall efficacy of 77-8%, and 93-4% against the severe symptomatic disease. The Covaxin is also reported to have good efficacy (65.2%) against Delta (B.1.617.2) variant of SARS-CoV-2 (8). The Covishield reported to have efficacy of 81% with two doses at 12 weeks apart (9).

The SARS CoV-2 infection (Breakthrough SARS CoV-2 infection) may dampen the efforts towards pandemic control by breaking public confidence on COVID vaccines. The breakthrough COVID infections are defined as the detection of SARS CoV 2 RNA or antigen in the respiratory samples of an individual 14 days after fully vaccination (10,11). Reports and studies of breakthrough SARS CoV-2 infections and COVID-19 have emerged recently (12–16). The breakthrough infections following COVID-19 vaccination is a global concern both clinically and epidemiologically. The breakthrough infection may show high transmissibility, virulence, and may result in higher morbidity and mortality. None of the COVID-19 vaccine developed till date has proven 100% efficacy. So, a fraction of population still remains susceptible to SARS CoV-2 despite full vaccination. The SARS CoV-2, especially the variants, also evades the immunity offered by the vaccines in some individuals (17). Frequent exposure of HCW and newer variants of SARS CoV-2 could be the reasons behind breakthrough infections (11,18).

A recent report released by Government of India in mid-April 2021 stated that nearly 0.04 per cent of people who have taken the second dose of COVAXIN have tested positive for Covid-19 (19). This data is reported passively by the beneficiaries and was collected when there was no acute surge in no of COVID-19 cases in India so there are chances of underreporting. A couple of published research from India have reported breakthrough SARS CoV-2 infection among the health care workers in hospital setting, albeit with smaller sample sizes. During the second wave of the pandemic many health care professionals and workers in our institute developed the SARS CoV-2 infection and COVID-19 diseases. We carried out this project to help prevent the SARS CoV-2 infections and diseases among the vaccinated individuals. The current project has two objectives; first, to estimate the proportion of breakthrough COVID-19 infection among completely vaccinated individuals and second, to study the clinico-epidemiological profile of breakthrough COVID-19 infections among them.

## Methods

**Study design, duration, ethical consideration**: We adopted a cross-sectional analytic design during the months of May and June 2021 to carry out this study. The study has been approved by Institute Ethics Committee, All India Institute of Medical Sciences (AIIMS), Patna (Ref. No. AIIMS/Pat/IEC/2020/705).

**Study setting**: We conducted this study at AIIMS, Patna, Bihar, India. AIIMS, Patna is one of the Institution of National Importance in India, and also one of the COVID Vaccination Centre (CVC) in Bihar, India. The CVC, AIIMS, Patna administers Covaxin to the eligible individuals in a phased manner as per the order issued by Government of India. The beneficiaries included all the healthcare-workers (HCW), the frontline-workers (FLW) and the individuals aged 45 years and above. We conducted this study among the vaccinated beneficiaries from AIIMS, Patna CVC. Till 10^th^ of May 2021, a total of 8,566 and 5,406 beneficiaries, which included HCWs, FLWs and people above 45 years of age, had been vaccinated by using COVAXIN for first and second dose respectively.

**Study population, inclusion and exclusion criteria**: We considered all the beneficiaries vaccinated fully (have received two doses) from the AIIMS, Patna, CVC till May 10^th^, 2021. We included the beneficiaries vaccinated with second dose of COVAXIN at the CVC and completed 14 days period post vaccination as on the date of telephonic interview (between last week of May to second week of June 2021). Beneficiaries not answering the phone call even after two extra attempts on two consecutive days were excluded. The beneficiaries who did not have mobile of their own couldn't be accessed, and hence were excluded.

**Sample size and sampling technique**: Assuming the proportion of breakthrough COVID-19 infections to be 15.9% (12), the minimum sample size was estimated to be 2434 at absolute precision of 1.59% (relative precision of 10%) and non-response rate of 20% using OpenEpi software version 3.2. The vaccinated beneficiaries list is maintained at the CVC along with their contact information which included residential address and mobile number. We adopted the systematic random sampling technique with sampling interval of 2 to select the participants for our study. The first participant was selected between 1^st^ and 2^nd^ beneficiary randomly by flipping the coin. Then every alternate person was selected for the study. We included a total of 2703 participants in our study.

**Study tool**: The information was collected using a pre-tested semi-structure questionnaire encrypted in Epicollect5 data capturing tool by trained investigators. Epicollect5 is a free mobile and web-based application for easy data collection, storage and retrieval (20). The data collectors called the potential participants and enrolled them after obtaining informed consent telephonically. The questionnaire was administered by telephonic interview and the information was collected in the Epicollect5. The Questionnaire included socio-demographic details, vaccination Details, COVID-19 appropriate behaviour details post 2nd dose of the vaccination, breakthrough COVID-19 infection details and post COVID-19 health status details of the vaccinated beneficiaries post 14 days of second dose of COVAXIN a dministration.

**The following operational definitions are used:**

**Completely/fully vaccinated**: received the 2^nd^ dose and has crossed 14 days following it

**Breakthrough COVID-19 infection following vaccination (BCV)**: tested positive for SARS CoV-2 RNA or antigen in respiratory sample or diagnosed to have COVID-19 using imaging methods after 14^th^ day following receipt of 2^nd^ dose

**BCV based on signs and symptoms suggestive of COVID-19**: individual who have developed signs & symptoms strongly suggestive of COVID-19 after 14^th^ day following receipt of 2^nd^ dose. We have done analysis separately for the BCV based on signs and symptoms, and have presented the same in the supplementary tables 1 & 2.

**Table 1:** Socio-demographic details of the participants and breakthrough COVID-19 infections following vaccination (BCVs), N=2703

Socio-demographic characters	Group	Frequency N (%) *	Confirmed BCV	

			N (%) #	95% CI
Over all		2703	274	9.1, 11.4
			(10.1)	
Gender	Male	1785	185	9.0, 11.9
		(66.1)	(10.4)	
	Female	918 (33.9)	94 (9.7)	8.4,12.4
Age (in years)	< 30	1099	137	10.6,
		(40.7)	(12.5)	14.6
	30 – 59	1061	113	8.9, 12.7
		(39.3)	(10.7)	
	≥ 60	543 (20.0)	24 (4.4)	2.9, 6.6
Education level	No formal education	29 (1.1)	0 (0.0)	0.0,14.6
	Studied up to class 10^Th^	459 (17.0)	21 (4.6)	2.9,7.0
	Studied beyond class 10^th^	2215	253	10.4,12.8
		(81.9)	(11.4)	
Nature of work engaged in	Unemployed	724 (26.9)	47 (6.5)	4.8,8.6
	Employed mainly in outdoor work	390 (14.5)	22 (5.6)	3.7,8.5
	Employed mainly in indoor work	1585	205	11.3,14.8
		(58.6)	(12.9)	
Residence	Urban	2103	208 (9.9)	8.7,11.3
		(78.0)		
	Semi-urban	457 (16.9)	51 (11.2)	8.5,14.5
	Rural	138 (5.1)	15 (10.9)	6.4,17.6
Diabetes Mellitus (DM)	Yes	298 (11.0)	17 (5.7)	3.5,9.1
	No	2405	257	9.5,12.0
		(89.0)	(10.7)	
Co-morbid conditions other than DM	Yes	332 (12.3)	24 (7.2)	4.8,10.7
	No	2371	250	9.4,11.9
		(87.7)	(10.5)	
Beneficiary Category	HCW	1453	193	11.6,15.2
		(53.8)	(13.3)	
	FLW	508 (18.8)	48 (9.4)	7,1,12.4
	Others$	742 (27.4)	33 (4.4)	3.1,6.3
Adequate COVID appropriate behaviour@	Yes	877 (32.5)	101 (11.5)	9.5,13.9
	No	1826	173 (9.5)	8.2,11.0
		(67.5)		
Mask	Yes	1445	165	9.9,13.2
		(53.5)	(11.4)	
	No	1258	109 (8.7)	7.2,10.4
		(46.5)		

* Colum percentage

# Row percentage

$ others included senior citizen and individuals aged 45–59 years

HCW = Health care worker; FLW = Frontline workers

@ based on four items on COVID appropriate behaviours (explained in the methods)

**Table 2:** Risk factors for confirmed BCVs, N=2703

Risk factor	Categories	Confirmed BCV	

		AOR (95% CI)	P value
Gender	Male	1.08(0.81–1.42)	0.612
	Female	1	
Age (in years)	< 30	1.15(0.52–2.53)	0.734
	30–59	1.12(0.53–2.37)	0.759
	≥ 60	1	
Education level	Beyond class 10	1.78(1.06–2.97)	0.028
	Up to class 10	1	
Occupation	Employed mainly in outdoor work	0.66(0.36–1.22)	0.186
	Employed mainly in indoor work	1.41(0.96–2.05)	0.080
	Unemployed	1	
Beneficiary category (exposure level)	Health care worker	3.04(1.50–6.15)	0.002
	Front line worker	2.53(1.19–5.39)	0.016
	Others	1	
Residence	Semi-urban	1.14(0.82–1.58)	0.448
	Rural	1.72(0.95–3.12)	0.076
	Urban	1	
Diabetes Mellitus	Present	1.19(0.64–2.20)	0.577
	Absent	1	
Other chronic co-morbidities	Present	1.57(0.91–2.69)	0.104
	Absent	1	
COVID-19 appropriate behaviour	Adequate	1.03(0.79–1.35)	0.806
	Inadequate	1	

BCVs=Breakthrough COVID-19 infections following full vaccination, AOR=Adjusted Odds ratio

**Bio-statistical analysis**: The information collected was downloaded from Epicollect5 in an MS Excel sheet and analysis was performed using IBM SPSS version 20 (IBM Corp. Released 2011. IBM SPSS Statistics for Windows, Version 20.0. Armonk, NY: IBM Corp.) and the Openepi software version 3.2 (21). Descriptive analyses were conducted to describe the socio-demographic characteristics, Vaccination Details, COVID-19 prevention appropriate behavior details post 2nd dose, Breakthrough COVID-19 Details and Post COVID-19 health status Details. The quantitative variables were described using mean, standard deviation (SD) and range. The categorical variables were expressed as proportions. Association between categorical variables with the COVID-19 status was assessed using Chi Square test and Fisher Exact Test. A multivariable model for logistic regression was developed to identify the independent risk factors for breakthrough COVID-19 among vaccinated individuals. For logistic regression model, age was categoried into three categories (< 30 years, 30–59 years and ≥ 60 years), education into two categories (no formal education/studies up to class 10, studied beyond class 10), occupation into three categories (unemployed, employed and involved mostly in indoor works, and employed and involved mostly in outdoor works), and beneficiaries into three categories (health care worker, frontline worker, other [elderly & 45–59 years]). The COVID appropriate behaviours included use of mask, avoidance of gatherings/crowded places, social distancing and hand hygiene (washing and/or sanitization). A person practicing three out of the four COVID appropriate behaviour always or most of the time was considered to have adequate COVID appropriate behaviour. A p-value of *<* 0.05 was considered statistically significant (two-sided tests).

## Results

**General characteristics of study participants**: The demographic details of the study participants are shown in Table 1. The majority of the participants were male (66%). Maximum participants (40.7%) aged less than 30 years. Nearly 60% of the participant's job involved activities which are primarily indoor. The majority of the participants reside in urban areas (78%) and were health care workers (HCW) by profession. An 11% of the participants reported to have diabetes mellitus (DM) while 12.3% had at least one chronic morbidity other than DM. Despite of the fact that majority of the participants (53.5%) were using mask while stepping out of home, only 32.5% had adequate COVID appropriate behaviour following their vaccination.

**Figure 1:**
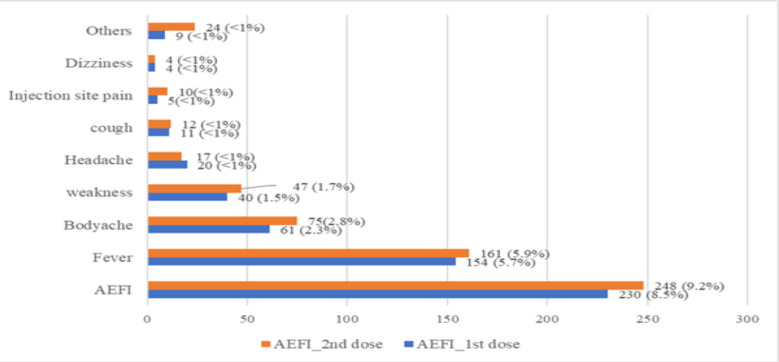
*AEFI following vaccine doses among the participants from CVC, AIIMS, Patna (N=2703)* AEFI=Adverse events following immunization, CVC=COVID-19 vaccination centre, AIIMS=All India Institute of Medical Sciences

**Figure 2:**
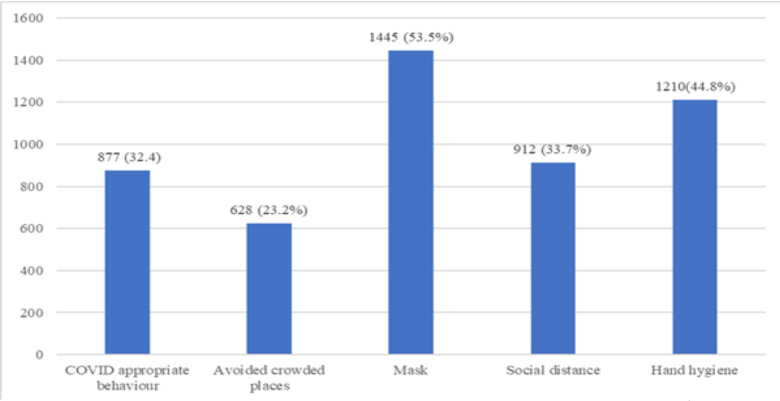
*Adequate COVID appropriate behaviours adopted by the participants following 2^nd^ dose of COVID-19 vaccines, N=2703;* COVID=Corona virus disease

**Details of vaccination**: The CVC, AIIMS, Patna administers Covaxin vaccine in two standard doses at 28 weeks apart minimum. The mean (standard deviation) gap between 1^st^ and 2^nd^ dose of vaccination was 31.7±7.3 days (ranged from 28 – 117 days). About 178 (6.6%) and 112 (4.1%) of the participants felt the CVC was overcrowded/social distancing not followed and that there was delay in verification of documents for vaccination respectively. Less than 10% participants reported adverse events following immunization with Covaxin; the most common being fever followed by body ache, weakness and headache (Figure 1). Post-vaccination only 32.5% had adopted adequate COVID appropriate behaviours (Figure 2).

**Estimates of breakthrough COVID-19 infections following vaccination (BCV)**: Details of BCV among participants based on their characteristics is provided in the Table 1. The estimate of confirmed BCV was found to be 10.1% (95% CI: 9.1%, 11.4%). The BCV was more among males (10.4%) than females (9.7%). The confirmed BCV was highest among the individuals aged ≤29 years (12.5%).

The estimate of BCV based on symptoms suggestive of COVID-19 was found to be 12.7% (95% CI: 11.4%, 13.9%). It was similar among males and females (Females: 12.9%; Males: 12.5%). It was also more among individuals aged ≤29 years (15.0%). The mean (standard deviation) duration between 2^nd^ dose of vaccine and testing positive for SARS CoV-2 was 41.9 ± 15.2 days and ranged from 14 to 87 days.

**Figure 3:**
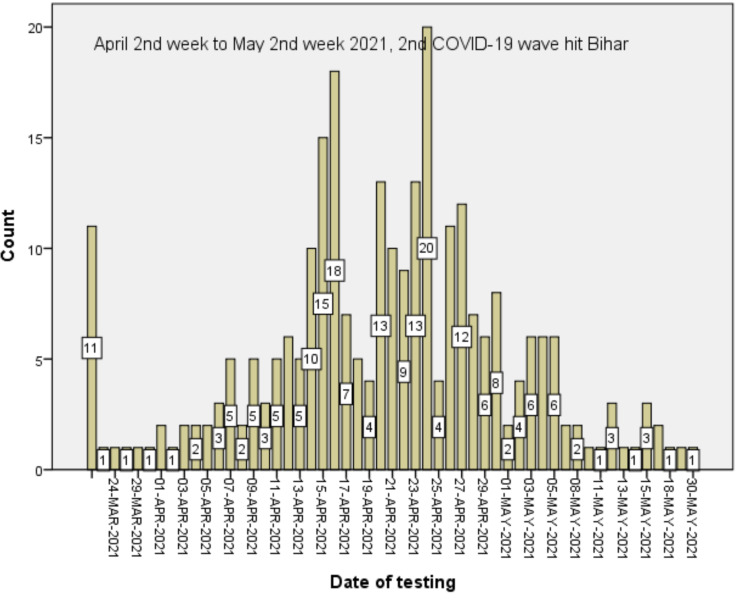
*Distribution of the BCVs among the full vaccinated individuals from CVC, AIIMS, Patna* BCVs=Breakthrough COVID-19 infections following full vaccination, CVC=COVID-19 vaccination centre, AIIMS=All India Institute of Medical Sciences

**COVID-19 appropriate behaviour and clinical details of the BCV**: Nearly half (274, 49.9%) of the 549 participants investigated for COVID-19 were confirmed to be COVID-19 positive. The various reasons for investigation were presence of symptoms (300), contact with a known COVID patient (180), Others (66), No response (3). The investigations performed were RT-PCR (520), rapid antigen (24), HR-CT (2), Other (1), Could not recall (2). The majority of the confirmed BCVs occurred during the second of wave of COVID-19 pandemic in India (Figure 3). A total of 223/274 (81.4%) confirmed BCVs were symptomatic. The symptoms experienced by them were fever (200), dry cough (166), body-ache (117), headache (88), loss of sense of smell (79), loss of sense of taste (68), running nose (49), nasal blockage (28) breathlessness (41), passing loose stool (13) and chest pain (12). More than 90% (251/274) were home isolated and only 8% (22/274) received hospital management, for one person treatment details not available. Only 3 cases needed intensive care unit (ICU) transfer. There is no mortality among the BCV cases. Tiredness (23/274) and cough (16/274) were common complaints reported by the participants post-recovery from the BCV. Nearly 95% (248/262, 12 did not respond) of the individuals with BCVs reported improvement in health after the diagnosis.

**Risk factors for BCV**: The quantification of risk for developing breakthrough COVID-19 infection following vaccination is presented in table 2. The participant's diabetes mellitus status, beneficiary category, use of mask while stepping out of house, age, education level and occupation were found to have significantly association (p value < 0.05) with the BCV among the vaccinated individuals. Being health care worker and frontline worker increased the risk of contracting the SARS CoV-2 infection independent of other factors. The HCW and FLW were at higher risk of developing confirmed BCV by 3 times and 2.5 times respectively compared to general population. Education beyond class 10 was also found to have increase the risk of BCV independently (Table 2). Other factors were not found to be an independent risk factors for developing BCV. However, the quantification of risk for other factors and their significant level is depicted in the Table 2.

## Discussion

The BCV is an important concern at the time when India is rolling out the largest COVID-19 vaccination campaign to all the citizens in India. It is highly important to be informed about the occurrence of BCV and to plan for preventive and rehabilitation measures to bring back the public confidence on vaccination at one hand, and to protect the vaccinated individuals at other hand. Researches around the world have reported BCVs, and that is a concern in fight against the COVID-19 pandemic.

In our study, about 10% of the participants had confirmed BCVs. A study from a hospital in Delhi, India has reported 15.9% of the fully vaccinated HCW to have COVID-19. (12) In our study, 13.3% of the HCW have reported BCVs. Teran et al reported about 3.5% of skilled nurses to have BCVs in a hospital from United States (US). (22) Another study from California, US reported BCVs of less than 1% among the HCW in a hospital. (23) A study from Greece reported 1.2% of BCV among HCW in a hospital. (24) In a study among miners in French, Papua New Guinea, 15 out of 25 fully vaccinated individuals were found to have COVID-19. (25) There are multiple reasons for occurrence of BCVs. First, none of the vaccine used for COVID-19 are 100% effective. The phase III trials of Covaxin reported vaccine efficacy of 78%. That means, still, a section of fully vaccinated population is susceptible to SARS CoV-2 infection. The new variant of SARS CoV-2 may evade the protection offered by the vaccine. (17) The viral load among some fully vaccinated individuals is still found to be comparable to that of unvaccinated and partially vaccinated individuals. (26) These BCVs not only pose a threat to colleague and peers but also to the family members through horizontal transmission.

The BCVs of 10% means rest 90% fully vaccinated individuals are protected. This needs to be interpreted carefully, as, some of the 90% would have developed immunity through exposure to natural infections. BCV was more common among the males, the younger individuals, the individuals who are mostly involved in indoor activities, and the HCW & FLW. This may be due to higher rate of exposure to SARS CoV-2 than their counterparts. A nationwide data from US reported 63% of the BCV cases to be female HCWs. (27) A study from Kerala also reported about 66% of the BCV to be female HCWs. (28) Individuals despite practicing adequate COVID appropriate behaviour has slightly higher risk of contracting BCV although not found statistically significant. The social desirability bias could explain this finding. Despite this contradictory finding, we recommend practicing of COVID appropriate behaviour even after full vaccination in line with the World Health Organization (WHO) recommendations.

Although the majority of the BCV cases were symptomatic in our study, less than 10% required hospitalization and no death was reported among the BCV cases. A couple of studies from Kerala, India also has reported similar findings with no deaths. (28,29) Similar symptoms, as ours, post recovery of BCVs have been reported by Niyas et al. Only one third of the BCV cases among the skilled nurses in a hospital from US were symptomatic. (22) According to CDC, out of the total BCVs reported till April 2021, 27% were asymptomatic, 10% required hospitalization and 2% died. (27) More than 95% of BCV cases improvement in health status in our study. Only few cases static perception of the health following BCVs and one person reported worsening of health.

The sample size of the study is large and adequate, and probably the first study of its kind from India. We could estimate the proportion of BCV following vaccination with the Covaxin. Vaccination being done in a single centre and that is in our institute, the probability of contracting SARS CoV-2 infection is less. The staff involved in vaccination are well trained and ensure safe injection practices for vaccination. Except rare situation, social distance and wearing mask are ensured at our vaccination centre. We have taken care to ensure recruitment of right person, enrolment of individuals only after 14 days of vaccination. The protective effect of the vaccine is established only after 14 days of administration. We have used Epicollect5 for ensuring quality of data collected.

Our study also has some limitations. Being a telephonic survey, the chances of reporting and recall bias can't be completely ruled out. We have tried these biases by ensuring uniform information collection by trained data collector (resident doctors and interns) and allowing sufficient time to the participants for their response. Since recall period was less than six months and COVID-19 is a major event, the chances of recall bias is assumed to be less. This is single vaccination centre study and more than half the participants are health care workers who have inherence higher risk of contracting SARS CoV-2 due to high-risk exposure. The estimates of BCV in other vaccination centre will be different and probably less than our study due to lower proportion of HCW beneficiaries.

In conclusion, one in every ten fully vaccinated individuals can contract BCVs. The HCW and FLW have 3 times and 2.5 times higher risk of getting BCVs than general population. Most of the BCVs occurs during the acute surge of COVID-19 cases in the community. The BCVs are not very serious and did not result in death.

Based on the findings from this study, we recommend that people need to practice adequate COVID appropriate behaviours even after full vaccination. The high-risk group (HCW & FLW) need to practice the COVID appropriate behaviour routinely and aggressively. The benefits of vaccination is far greater and the public should not be distracted by the reports of BCVs. Not to panic about the BCVs, as recovery is well and none results in death or long-term disability.
